# A new efficient approach to fit stochastic models on the basis of high-throughput experimental data using a model of IRF7 gene expression as case study

**DOI:** 10.1186/s12918-017-0406-4

**Published:** 2017-02-20

**Authors:** Luis U. Aguilera, Christoph Zimmer, Ursula Kummer

**Affiliations:** 1 Department of Modeling of Biological Processes, COS Heidelberg / Bioquant, Heidelberg University, Im Neuenheimer Feld 267, Heidelberg, 69120 Germany; 2 BIOMS (Center for Modeling and Simulation in the Biosciences), Heidelberg University, Im Neuenheimer Feld 267, Heidelberg, 69120 Germany

**Keywords:** IRF7, IFN, Stochastic models, Parameter estimation

## Abstract

**Background:**

Mathematical models are used to gain an integrative understanding of biochemical processes and networks. Commonly the models are based on deterministic ordinary differential equations. When molecular counts are low, stochastic formalisms like Monte Carlo simulations are more appropriate and well established. However, compared to the wealth of computational methods used to fit and analyze deterministic models, there is only little available to quantify the exactness of the fit of stochastic models compared to experimental data or to analyze different aspects of the modeling results.

**Results:**

Here, we developed a method to fit stochastic simulations to experimental high-throughput data, meaning data that exhibits distributions. The method uses a comparison of the probability density functions that are computed based on Monte Carlo simulations and the experimental data. Multiple parameter values are iteratively evaluated using optimization routines. The method improves its performance by selecting parameters values after comparing the similitude between the deterministic stability of the system and the modes in the experimental data distribution. As a case study we fitted a model of the IRF7 gene expression circuit to time-course experimental data obtained by flow cytometry. IRF7 shows bimodal dynamics upon IFN stimulation. This dynamics occurs due to the switching between active and basal states of the IRF7 promoter. However, the exact molecular mechanisms responsible for the bimodality of IRF7 is not fully understood.

**Conclusions:**

Our results allow us to conclude that the activation of the IRF7 promoter by the combination of IRF7 and ISGF3 is sufficient to explain the observed bimodal dynamics.

**Electronic supplementary material:**

The online version of this article (doi:10.1186/s12918-017-0406-4) contains supplementary material, which is available to authorized users.

## Background

Computer models contribute to the integrative understanding of complex molecular processes in the cell. The most commonly used approach is deterministic modeling based on ordinary differential equations (ODEs). When the studied system comprises species with a low molecular count, stochastic formalisms, e.g. Gillespie’s algorithm that simulates trajectories and uses discrete molecule numbers are more appropriate [1]. In recent years, the use of stochastic models has substantially increased [2].

Considering stochasticity in biological systems has changed the quantitative and qualitative understanding obtained by previous deterministic models [3]. Examples of biological phenomena discovered by stochastic modeling include gene expression in burst-like patterns [4], productive or latent cell decision after HIV-infection [5], and the presence of oscillatory behavior induced by noise [6]. However, in contrast to the plethora of methods used to fit and analyze deterministic models [7–10], there are only very limited sets of methods available to do the same with stochastic models.

Thus, only recently, methods for parameter fitting of stochastic models have been developed and are still under development. Parameter estimation methods for stochastic models have been so far designed for single time course data [11–16]. For experiments leading to data distributions, they include the moment closure [17–19], and the comparison of experimental and simulation distributions [20–22]. Despite the current efforts, there are still major problems that limit the full applicability of those methods. The main drawback is the high computational execution time. To solve this problem novel efficient strategies to fit stochastic models are needed.

In the following we introduce a new approach that is a variant of existing methods based on the comparison of distributions. Differently to the existing methods we use the experimental data to define a condition that the model must fulfill in the deterministic regime. This condition tests, if the deterministic steady states are in the close proximity to the modes of the experimental distribution. Only if the model fulfills this condition, the parameter set is evaluated in the stochastic regime. In this way, the algorithm directs the evaluation of parameter sets towards regions in parameter space with high potential to reproduce the experimental data. Additionally, this method applies to non-linear models and complex experimental data distributions.

To test the potential of this new method, we selected an open scientific question and real experimental data. We investigated the molecular mechanisms responsible for the experimentally observed bimodal dynamics of IRF7 expression after Interferon (IFN) stimulation.

It is well documented that in isogenic cell populations not all cells produce an antiviral response when infected by a virus [23]. The mechanisms behind this heterogeneity have been related to stochastic events in the signaling pathways responsible to elicit the antiviral response [24–26]. IFN is a cytokine that activates the JAK-STAT signaling pathway in virus-infected cells. The JAK-STAT signaling pathway induces the translocation of ISGF3 (a transcription factor) into the nucleus to directly activate the transcription of a set of several hundred IFN-stimulated genes (ISGs) [27]. IRF7 is an ISG with a central role in the immune response [28, 29]. Recently, it was observed that after IFN stimulation murine fibroblasts show a switch-like pattern of IRF7 expression, which is reflected at the population level as bimodality [30].

We developed a model to describe the observed bimodal dynamics of IRF7 expression after IFN stimulation. We hypothesize that the binding of IRF7 and ISGF3 to the IRF7 promoter is the mechanism responsible for this bimodal behavior. Our simulation results reproduced IRF7 switch-like dynamics at single cell level, and bimodality was achieved at the population level. Hereby, we used the newly developed method to fit the model and correctly reproduce the experimental data. Our results allow us to conclude that the IRF7 promoter activation by the combination of IRF7 dimers and ISGF3 is sufficient to quantitatively explain the observed IRF7 bimodal dynamics.

## Methods

### Experimental data

Published experimental data were produced by Rand et al. [30]. The data described the expression of IRF7 after IFN- *β* stimulation in a population of murine NIH3T3 fibroblasts. Rand’s experiments were done in the following way: First, cells were transfected with a BAC (Bacterial Artificial Chromosome) containing IRF7 and reporter mCherry genes fused, subsequently cultures were treated with different concentrations of murine IFN- *β*. For illustrative purposes we selected the treatment with 150U of IFN- *β*, a concentration where bimodality is prevalent. Then, fluorescence in the cultures was monitored using flow cytometry at different time points during 48 hours.

### Numerical methods

In the deterministic regime the model was simulated using symbolic methods in Matlab [31] and/or using the LSODA algorithm in COPASI 4.11 [32]. Stability was calculated by making the right-hand side of the differential equations equal to zero and subsequently by finding the roots of the system using function *solve* in Matlab. Eigenvalues were calculated using function *eig* in Matlab. For solving the model under stochastic dynamics we used the Gibson and Bruck algorithm [33] coded in COPASI. The random search and genetic algorithm were coded in Matlab. Raw experimental data was analyzed using the function *FCS data reader* coded in Matlab [34]. The modes in the distributions were calculated using the function *PeakFinder* coded in Matlab [35]. The source code of the project can be accessed via: https://sourceforge.net/projects/irf7-bimodaldynamics/.

## Results

### New algorithm to fit stochastic biological models

#### Comparing experimental and simulation distributions

The measurements of fluorescence were made comparable with the corresponding observable chemical species *S* ^*o*^ in the model by a function *h* that maps state *S* ^*o*^(*t*_*i*_) to observation *S* ^*†*^(*t*_*i*_) as follows:

1 $$ S^{\dagger}({t}_{{i}}) = h (S^{o}({t}_{{i}})),  $$

at each measurement time point *t*_*i*_, *i*=1,…,*n*.

Subsequently, a process to compare distributions was developed based on [20]. First, considering a specific set of parameter values *θ*={*θ*_1_,…,*θ*_*d*_}, we performed *ns* repetitions of the stochastic simulations ***s***(*t*_*i*_)={*s*_1_(*t*_*i*_),…,*s*_*ns*_(*t*_*i*_)}. The total of those stochastic simulations were used to build histograms with a fixed number of bins *L* for each *t*_*i*_. Subsequently, the (discrete) probability density function (PDF) of the simulations *P*_*s*_(***s***(*t*_*i*_),*θ*,*b*_*l*_) were computed using the center of each bin *b*_*l*_, for *l*=1,…,*L*, and the normalized form of the histograms. On the other hand, having *nm* repetitions of single cell experimental data ***m***(*t*_*i*_)={*m*_1_(*t*_*i*_),…,*m*_*nm*_(*t*_*i*_)} histograms and PDFs *P*_*e*_(***m***(*t*_*i*_),*b*_*l*_) were built. Even though, complex formulas can be use to calculate the number of bins in an histogram, here *L* was kept constant for *P*_*s*_ and *P*_*e*_ and was calculated as $L = \sqrt {nm}$, this is a simple approach used by default by most programming languages (i.e. Matlab). Finally, an approach using the difference of squares was used as a metric to calculate the distance between *P*_*s*_(***s***(*t*_*i*_),*θ*,*b*_*l*_) and *P*_*e*_(***m***(*t*_*i*_),*b*_*l*_), as follows:

2 $$ F(\theta,\boldsymbol{m}) = \sum_{i=1}^{n} \sum_{l=1}^{L} (P_{e}(\boldsymbol{m}(t_{i}),b_{l}) - P_{s}(\boldsymbol{s}(t_{i}),\theta,b_{l}))^{2}.  $$

#### Algorithm implementation

The objective function *F*(*θ*,***m***) can be used to calculate a parameter estimate

3 $$ \hat{\theta} = \text{argmin}_{\theta} F(\theta,\boldsymbol{m}).  $$

As optimization usually requires plenty of function evaluations and each evaluation of *F* requires *ns* stochastic simulations, a direct optimization strategy is computationally unfeasible even for simple models. Therefore, we introduce a new strategy that selects good candidate parameters and only performs the stochastic simulations for these parameters.

In large systems where fluctuations can be discarded, the stochastic system can be reduced to the deterministic one [36, 37]. For this reason, in most cases the deterministic dynamics can be associated with a measure of central tendency in the PDFs obtained after the stochastic simulations. Using this reasoning we will introduce the strategy to efficiently estimate parameters for stochastic models making use of deterministic dynamics as an initial indicator.

Assuming that the experimental data is in equilibrium at the last measurement point *t*_*n*_, we used the modes from *P*_*e*_(***m***(*t*_*n*_),*b*_*l*_) as a central measure of tendency, denoting the modes by ***α***(*t*_*n*_)=(*α*_1_(*t*_*n*_),…,*α*_*q*_(*t*_*n*_)), with *q* being the total number of modes.

Then, using the ODE version of the model ($\dot {\boldsymbol {X}}(\theta,t)$) we determine its stability. Here we tested whether the system has stable steady states and denote them with $\boldsymbol {X}^{*}(\theta) = \left (X^{*}_{1}(\theta),\ldots,X^{*}_{ss}(\theta) \right)$, being *s* *s* the number of stable steady states. If the system has no stable steady state, it holds *s* *s*=0 and the vector ***X*** ^∗^(*θ*) is empty. The calculation of steady states can either be performed by solving $\dot {\boldsymbol {Y}}(\theta) = 0$ where $\dot {\boldsymbol {Y}}$ is the right hand side of the ODE system with ***X***(*θ*,*t*) replaced by ***Y***(*θ*). Solving this equation can be impossible for large systems. Alternatively, one can numerically solve the ODE systems until the flux is zero for all components. Varying the initial conditions leads to the different steady states. The stability of the system was calculated after determining the sign of the eigenvalues (*λ*<0), for stable steady states [38].

Having ***X*** ^∗^(*θ*) and ***α***(*t*_*n*_) we introduce the *deterministic precondition*:

4 $${} \begin{aligned} &\beta_{k}^{low} X_{k}^{*}(\theta) \leq \alpha_{k}(t_{n}) \leq \beta_{k}^{up} X_{k}^{*}(\theta),\\ \text{for}\ 1\leq& k \leq q,\ \text{and}\ 0 \leq \beta_{k}^{low} \leq1\ \text{and}\ 1 \leq \beta_{k}^{up}. \end{aligned}  $$

**Fig. 1:**
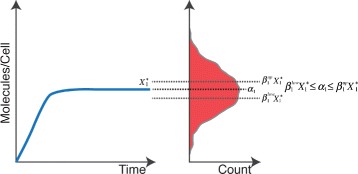
The deterministic precondition. Left side the deterministic dynamics of the system can be seen, here the observable variable evolves to a single stable steady state $X_{1}^{*}(\theta)$. This steady state can be calculated by finding the point in the system where the net flux is equal to zero $\dot {\mathbf {X}}(\theta) = 0$. At the right hand side of the plot, the PDF of the experimental data is shown. This PDF is rotated and the x-axis shows the count and the y-axis shows the bin values with units of Molecules/Cell. From both graphs it can be seen that the deterministic precondition is valid when $X_{1}^{*}(\theta)$ is inside the range defined by $\beta _{1}^{low} X_{1}^{*}(\theta)$ and $\beta _{1}^{up} X_{1}^{*}(\theta)$

Equation (4) means that the number of modes equals the number of stable steady states: *s* *s*=*q*, and that each of the stable steady states is close to its corresponding mode. Only, if this is fulfilled, we calculate stochastic simulations and evaluate the objective function *F*(*θ*,***m***). If the precondition is not fulfilled, we do not carry out stochastic simulations. In this case, we assign a high (bad) objective function value to direct the parameter search towards parameters that pass the deterministic precondition. A graphical representation of the deterministic precondition is given in Fig. 1.

At this point, it is important to notice that the deterministic precondition is not stating that the stable steady states form the ODE system are equal to the mean obtained by solving the stochastic system. Rather, the deterministic precondition tests if the deterministic steady state lies around a certain range.

5 $$ F_{\text{cond}}(\theta,\boldsymbol{m})\ = \ \left\{\begin{array}{ll} F(\theta,\boldsymbol{m}), &\beta_{k}^{low} X_{k}^{*}(\theta) \leq \alpha_{k}(t_{n}) \leq \beta_{k}^{up} X_{k}^{*}(\theta) -- ^{\prime\prime}\text{Condition passed}^{\prime\prime} \\ \infty, &\quad\text{else --} ^{\prime\prime}\text{Condition not passed}^{\prime\prime} \end{array}\right.  $$

Our extended objective function with the deterministic check reads as:

and the estimate is defined as:

6 $$ \hat{\theta}_{\text{cond}} = \text{argmin}_{\theta} F_{\text{cond}}(\theta,m).  $$

**Fig. 2:**
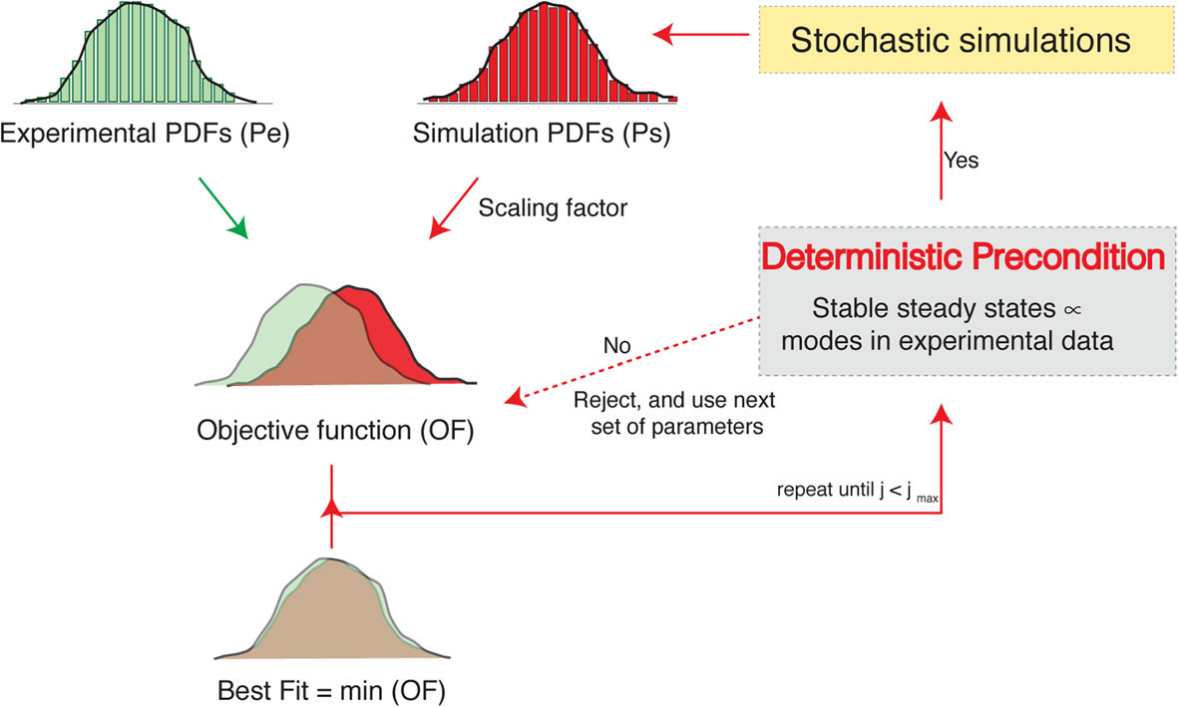
Algorithm to fit stochastic models to experimental data. The algorithm solves the model in the deterministic and stochastic regimes. A condition observed in the experimental data is defined. A set of parameter values is evaluated in the deterministic regime to test if the model reproduces this condition. If the condition is met the stochastic simulation is performed. Otherwise, the parameter values are rejected. The PDF obtained from the experimental data is compared with the PDF obtained after running the stochastic simulations. This comparison is made using a difference of squares as an objective function. This process is repeated until evaluating a total number of parameter sets or after a termination criterion is met. The parameter set that best reproduces the experimental data is given by the minimum value obtained after the iterative evaluation of the objective function

As previously discussed, our method is based on the central assumption that the modes in the experimental data are related to the stable steady states in the deterministic mathematical model. If this assumption should fail, either of the following could happen: a) The method accepts a parameter that passed the deterministic precondition but does not show a good agreement of the distributions. If it does not show good agreement, the objective function *F*(*θ*,***m***) will show a high value which means that this parameter does not lead to a good fit. Therefore, this case leads to a loss of computational time, but not to wrong results and it overall is still less expensive than evaluating every parameter set. b) We reject a parameter that fails the deterministic precondition but would have lead to a good fit. In this case, we lose indeed a good parameter. This shows that our method is obviously a heuristic strategy that does not guarantee to find the exact global minimum, but this is true for almost any optimization strategy. The algorithmic steps are depicted in Fig. 2.

#### Parameter estimation methods

The deterministic precondition was first implemented using a random search algorithm. Random search is a global optimization strategy that tests random combinations of parameter values. The successful output in this method is dependent on the total number of evaluated parameter sets [39]. The more evaluated parameter values, the higher the probability to find the global minimum. The pseudo-code for the random search is given in Algorithm 1.



The second implementation of the deterministic precondition was using the more directed Genetic Algorithm. Genetic Algorithm mimics evolution and is based on reproduction and selection. This algorithm is made of a population of individuals (parameter sets), and each contains a genome that is defined by the number of parameters to optimize. The individuals are ranked after solving the objective function, and a population of parental individuals is selected according to an elitism rate (*ε*). New individuals (offspring) are generated by pairing and recombining the parental genomes (cross-over). Variability is introduced in the population by adding mutations in the new individuals according to a given mutation rate (*μ*). By the continuous process of selecting the best parameters after each generation, the algorithm evolves towards the regions in the parameter space that reduce the values in the objective function. The pseudo-code for the Genetic Algorithm is given in Additional file 1.

## Identifying parameters for a constitutive gene expression circuit with in-silico data

**Fig. 3:**
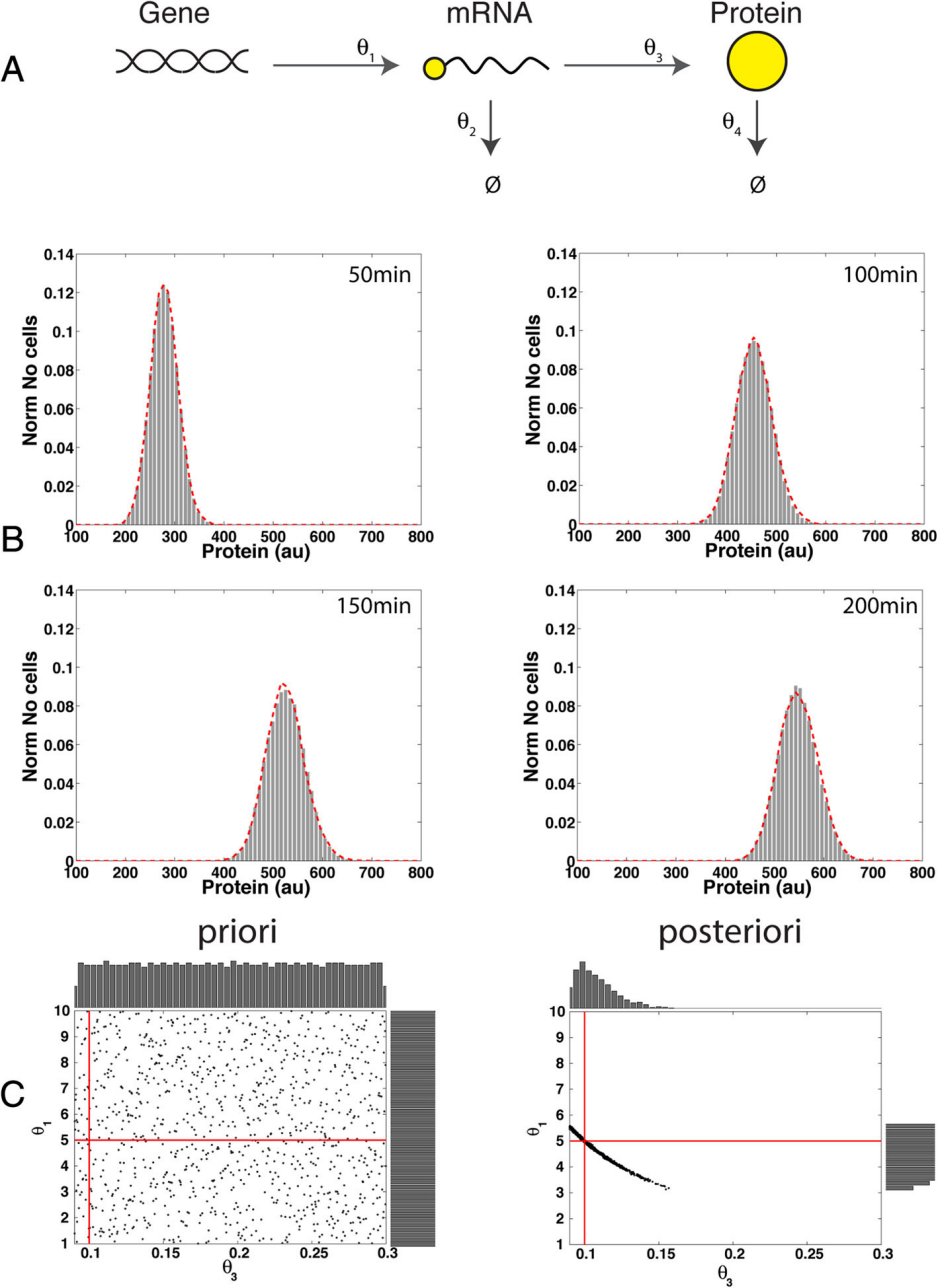
Parameter estimation using a constitutive gene expression circuit and in-silico data. **a** Diagram of a gene with constitutive expression. The mRNA is produced at constant rate *θ*_1_ and is degraded at constant rate *θ*_2_, the proteins are produced at constant rate *θ*_3_ and are degraded with *θ*_4_. **b** Comparison between in-silico data and model dynamics using the true parameters *θ* ^*o*^=(5,0.03,0.1,0.03) and the estimated parameters $\hat {\theta } = (5.086,0.03,0.098,0.03)$. A distribution with the in-silico data is given in grey. In red is given the model dynamics. **c** Comparison between a priori and a posteriori parameter distributions. The true parameters are represented by the intersection of the red lines. In the priori distribution it can be observed that 1000 parameters are randomly distributed in the parameter space. The posteriori distributions were calculated by running 1000 independent random searches with 1000 parameters each, and by plotting the best parameter value selected by the algorithm. In the posteriori distribution it is plotted only the parameters that passed the deterministic precondition, and it can be observed that those parameter estimates are in a region close to the true parameter values

To prove the functionality and benefits of the new proposed algorithm, we first applied it to a simple example where the parameters are known a priori. The model describes the stochastic dynamics of two variables, protein and mRNA of a gene with constitutive expression [40], a graphical representation of the constitutive gene expression circuit is given in Fig. 3-a. The model is described by the following reactions:

7 $$ \varnothing \stackrel{\theta_{1}}{\longrightarrow} mRNA,  $$

8 $$ { mRNA \stackrel{\theta_{2}}{\longrightarrow} \varnothing },  $$

9 $$ {{ mRNA \stackrel{\theta_{3}}{\longrightarrow}}Protein},  $$

10 $$ { Protein \stackrel{\theta_{4}}{\longrightarrow} \varnothing}.  $$

To estimate the parameter values for this model we carried out the following procedure: First, we generated the in-silico data by selecting the following true parameter values *θ* ^*o*^=(5,0.03,0.1,0.03) and running 10000 independent stochastic simulations from which PDFs were built. Four observable time points were defined at 50 min, 100 min, 150 min and 200 min (see Fig. 3 b). From the PDFs a mode was obtained at *α*_1_(*t*_*n*_) = [543] a.u.(arbitrary units), where *t*_*n*_=200 min. Then, we defined the deterministic precondition using Eq. (4), and assigning minimum and maximum acceptance ranges by setting $\beta _{1}^{low}$ = 0.95 and $\beta _{1}^{up}$ = 1.05, respectively. A deeper analysis of the stability of the constitutive gene expression circuit is given in Additional file 2. To build simulated PDFs we used 1000 repetitions of the stochastic model, this number was empirically calculated according to Additional file 2. For illustrative purposes, we assumed that the values for the parameters responsible for the mRNA transcription and protein translation (*θ*_1_, *θ*_3_) were unknown, and the new algorithm was used to estimate those parameters. The deterministic precondition was applied using the RS strategy obtaining that the algorithm only evaluates stochastic dynamics in 3.1% of the tested parameter values, reducing in this way the total simulation time. Additionally, the complete algorithm was repeated 1000 times and histograms of the estimated parameter were computed to determine whether they are close to *θ* ^(0)^. As can be observed in Fig. 3-c the deterministic precondition reduces the evaluation of different parameters under stochastic dynamics by selecting only those parameters that are in a well-defined region in the proximity of *θ* ^(0)^. For this model, the main benefits of using the deterministic precondition was the reduction of the number of parameters evaluated under stochastic dynamics. This rejection of parameters was made in an area outside the true parameter values, and hence no difference in accuracy is expected in comparison with a method without using the deterministic precondition. A complete description of the analysis of the performance, accuracy and error for this example is given in Additional file 2. The model for the constitutive gene expression circuit can be obtained from BioModels database under reference MODEL1608100000.

## Mathematical model for IRF7 expression dynamics

**Fig. 4:**
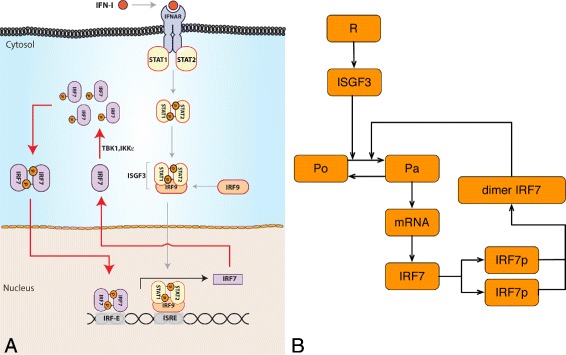
IRF7 gene expression circuit. **a** In viral infected cells IRF7 gene expression is induced after IFN stimulation by the JAK-STAT signaling pathway. The IRF7 promoter activation is governed by the binding of IRF7 dimer and ISGF3 to the promoter DNA binding sites (ISRE and IRFE). IRF7 promoter activation leads to the transcription of IRF7 mRNA and subsequent its translation to produce the IRF7 protein. Notice that the IRF7 production and subsequently phosphorylation and dimerization leads to the binding to its own DNA binding site, resulting in the production of more IRF7, making in this way a positive feedback loop. **b** A simplified system representing the IRF7 gene expression dynamics is given. Here, the promoter transitions between active/basal states (Pa/Po), the gene transcription and translation processes are represented as solid *black lines* as well as the feedback loop in the system

Subsequently we applied our algorithm to a real problem with flow cytometry data. Here we studied the dynamics of murine IRF7 gene expression upon IFN stimulation. For this reason, we developed a model that comprises known key components and feedback mechanisms. The overall system describes the active IRF7 promoter (*Pa*) by the binding of IRF7 dimer and ISGF3 to the DNA binding sites ISRE and IRFE, respectively [41], the transcription and translation of IRF7, and its subsequent phosphorylation and dimerization. IRF7 protein binding to the IRFE binding site in the promoter results in the production of more IRF7 protein, constituting a positive feedback loop [42]. A graphical representation of the IRF7 gene expression dynamics is given in Fig. 4.

### Chemical reactions

The developed model consists of 7 species and 13 reactions (reactions (11) to (23)). In the model, reaction (11) describes the IFN activation of the JAK-STAT signaling pathway. For the description of this reaction and according to [30] a saturable function was used. Downstream reactions of the pathway were lumped in reaction (12), so that the same variable was used to describe the output of the JAK-STAT signaling pathway, namely ISGF3, in this highly simplified model. Reaction (13) describes the IRF7 promoter activation by the binding of IRF7 dimer and ISGF3 to ISRE and IRFE, respectively. Subsequently, we incorporated IRF7 mRNA transcription by the active promoter, and to a lesser extent by the basal promoter state, as reactions (14) and (15), respectively. Reaction (16) considers the translation of IRF7 mRNA to produce IRF7 protein. IRF7 protein is phosphorylated in reaction (17). Two phosphorylated IRF7 proteins form a IRF7 dimer in reaction (18). Finally, IRF7 promoter inactivation, degradation of mRNAs, ISGF3, IFN and IRF7 proteins are represented by reactions (19) to (23), respectively:

11 $$ {IFN \xrightarrow{f_{IFN}}2 IFN },  $$

12 $$ {IFN \xrightarrow{f_{ISGF3}} ISGF3 + IFN },  $$

13 $$ {\varnothing \xrightarrow{f_{Pa}} Pa},  $$

14 $$ { Pa \xrightarrow{f_{mRNA_{A}}}mRNA + Pa },  $$

15 $$ {\varnothing \xrightarrow{f_{mRNA_{B}}}mRNA},  $$

16 $$ { mRNA \xrightarrow{f_{IRF7}} IRF7 + mRNA},  $$

17 $$ { IRF7 \xrightarrow{f_{IRF7phos}} IRF7 phosp},  $$

18 $$ { 2 \cdot IRF7 phosp \xrightarrow{f_{IRF7dimer}}IRF7dimer},  $$

19 $$ { Pa \xrightarrow{f_{dPa}} \varnothing},  $$

20 $$ { IFN \xrightarrow{f_{dIFN}}\varnothing},  $$

21 $$ { mRNA \xrightarrow{f_{dmRNA}} \varnothing},  $$

22 $$ { IRF7dimer \xrightarrow{f_{dIRF7dimer}}\varnothing},  $$

23 $$ { ISGF3 \xrightarrow{f_{dISGF3}}\varnothing},  $$

The reaction rates are given in Table 1.

**Table 1:** Reaction rates considered in the model

Name	Definition
*f*_*IFN*_	$V_{IFN}\left (\frac {IFN^{n}}{k^{n}_{IFN}+IFN^{n}}\right)$
*f*_*dIFN*_	*k*_*dIFN*_·*I* *F* *N*
*f*_*ISGF*3_	*k*_*ISGF*3_·*I* *F* *N*
*f*_*dISGF*3_	*k*_*dISGF*3_·*I* *S* *G* *F*3
$f_{P_{a}}$	$k_{on}\left (\frac {ISGF3 \cdot IRF7dimer}{k_{aI3} \cdot k_{aI7} + k_{aI3} \cdot ISGF3 + k_{aI7}\cdot IRF7dimer + ISGF3 \cdot IRF7dimer}\right)(1-P_{a})$
$f_{dP_{a}}$	*k*_*off*_·*P*_*a*_
$f_{mRNA_{A}}$	*k*_*Active*_·*P*_*a*_
$f_{mRNA_{B}}$	*k*_*Basal*_(1−*P*_*a*_)
*f*_*dmRNA*_	*k*_*dmRNA*_·*m* *R* *N* *A*
*f*_*IRF*7_	*k*_*IRF*7_·*m* *R* *N* *A*
*f*_*IRF*7*phosp*_	*k*_*dIRF*7_·*I* *R* *F*7
*f*_*IRF*7*dimer*_	*k*_*IRF*7*dimer*_·*I* *R* *F*7*p* *h* *o* *s* *p*·*I* *R* *F*7*p* *h* *o* *s* *p*
*f*_*dIRF*7*dimer*_	*k*_*dIRF*7*dimer*_·*I* *R* *F*7*d* *i* *m* *e* *r*

### Mathematical equations

To evaluate the deterministic precondition we consider the corresponding ODEs (Eq. (24) to (30)):

24 $$\begin{array}{*{20}l} \frac{d \ IFN}{dt} &= f_{IFN}-f_{dIFN} \end{array} $$

25 $$\begin{array}{*{20}l}  \frac{d \ ISGF3}{dt} &= f_{ISGF3} - f_{dISGF3} \end{array} $$

26 $$\begin{array}{*{20}l}  \frac{d \ P_{a}}{dt} &= f_{P_{a}} - f_{dP_{a}} \end{array} $$

27 $$\begin{array}{*{20}l}  \frac{d \ mRNA}{dt} &= f_{mRNA_{A}} + f_{mRNA_{B}} - f_{dmRNA} \end{array} $$

28 $$\begin{array}{*{20}l}  \frac{d \ IRF7}{dt} &= f_{IRF7} - f_{IRF7phosp} \end{array} $$

29 $$\begin{array}{*{20}l}  \frac{d \ IRF7phosp}{dt} &= f_{IRF7phosp} - 2 \cdot f_{IRF7dimer} \end{array} $$

30 $$\begin{array}{*{20}l}  \frac{d \ IRF7dimer}{dt} &= f_{IRF7dimer} - f_{dIRF7dimer} \end{array} $$

The model for the IRF7 circuit can be obtained from BioModels database under reference MODEL1608100001.

### Fitting the stochastic IRF7 gene expression model to experimental data

All parameters of the above described model for the IRF7 gene circuit were fitted by using experimental data of IRF protein expression. In the model, a global quantity to describe the different forms of the IRF7 protein was defined as follows:

31 $$ IRFtotal (t)= IRF7(t) + IRF7phosp(t) + IRF7dimer(t),  $$

and this variable was mapped to IRF7^†^ (*t*_*i*_) flow cytometry measurements as follows:

32 $$ IRF7^{\dagger}({t}_{{i}}) = \varphi IRF7total({t}_{{i}}),  $$

where *φ* is a scaling factor.

Using the experimental data, PDFs were build and the modes in the distributions were determined using the PeakFinder function [35] obtaining two elements of *α*(*t*_*n*_) = [77, 1000] a.u. (arbitrary units), where *t*_*n*_ = 48 h. *φ* relates the values of fluorescence with the molecular count described by the mathematical model. Unfortunately, no calibration curve is provided with the data to calculate this parameter. For this reason, multiple values were tested for *φ* obtaining consistent results, for illustrative purposes we report *φ* = 1. We defined the deterministic precondition using Eq. (4), and assigning minimum and maximum acceptance ranges by setting $\beta _{k}^{low}$ = 0.95 and $\beta _{k}^{up}$ = 1.05, for *k* = 1, 2. The allowed ranges for the parameter values are given in Table 2. The deterministic precondition was introduced in two different optimization strategies, random search, and genetic algorithms. In both optimization strategies 1000 realizations of the stochastic simulations were performed if the parameter set fulfilled the deterministic precondition.

**Table 2:** Description of the parameter values

Name	Description	Range	Nominal	Units
*V*_*IFN*_	Maximum activation rate of the IFN pathway	[ 2.8,11.2]	6.135	(Molecules/Cell) ^∗^min
*n*	Hill coefficient	-	2	Dimensionless
*k*_*IFN*_	Saturation constant for the IFN pathway	[ 0.0022,0.0088]	0.0055	Molecules/Cell
*k*_*dIFN*_	Decay rate of the IFN pathway	[ 0.0232,0.0926]	0.0492	min^-1^
*k*_*ISGF*3_	Constant for ISGF3 production	[ 0.00012,0.00048]	0.0003	min^-1^
*k*_*dISGF*3_	Decay rate of the ISGF3	[ 0.00068,0.00272]	0.0017	min^-1^
*k*_*on*_	Promoter activation	[ 184.55,738.2]	522.59	min^-1^
*k*_*aI*3_	Constant of promoter activation by IFN	[ 7681.6,30727]	22687.02	Molecules/Cell
*k*_*aI*7_	Constant of promoter activation by IRF7	[ 13399,53597]	35281.99	Molecules/Cell
*k*_*off*_	Promoter inactivation	[ 0.00044,0.00176]	0.0013	min^-1^
*k*_*Active*_	IRF7 transcription rate by active promoter	[ 0.5402,2.161]	1.144	min^-1^
^*a*^ *k*_*Basal*_	IRF7 basal transcription rate	[ 0.0312,0.125]	0.0861	min^-1^
^*b*^ *k*_*dmRNA*_	Decay rate of mRNA	[ 0.029,0.116]	0.0715	min^-1^
^*c*^ *k*_*IRF*7_	Translation rate of IRF7	[ 14,56]	43.867	min^-1^
*k*_*dIRF*7_	Rate of IRF7 phosphorylation	[ 1.540,6.160]	3.877	min^-1^
*k*_*IRF*7*dimer*_	Rate of IRF7 dimerization	[ 0.235,0.94]	0.602	(Cell/Molecules)/min
*k*_*dIRF*7*dimer*_	Decay rate of IRF7 dimers	[ 0.209,0.836]	0.439	min^-1^
*φ*	Scaling factor	-	1	Cell/Molecules

^a^ *k*_*Basal*_ was calculated to be at least one order of magnitude smaller than *k*_*Active*_

^b^Degradation rates for the mRNA were calculated assuming a mRNA half-life in the order of minutes [50]

^c^Based on the average translation rate in NIH3T3 cells [49]

Using the random search strategy, we tested 10000 parameter sets from which less than the 1% passed the deterministic precondition and were stochastically evaluated. The reduction of parameter values allowed us to efficiently find a set of parameter values that reproduced the experimental data (the fitting for the random search algorithm is given in Additional file 3). A complete analysis of the performance of the use of the deterministic precondition with the random search algorithm is given in Additional file 4. To test the reproducibility of the estimated parameter values, the method was repeated 100 times obtaining well-defined parameter distributions that show the predominant values, see Additional file 5.

**Fig. 5:**
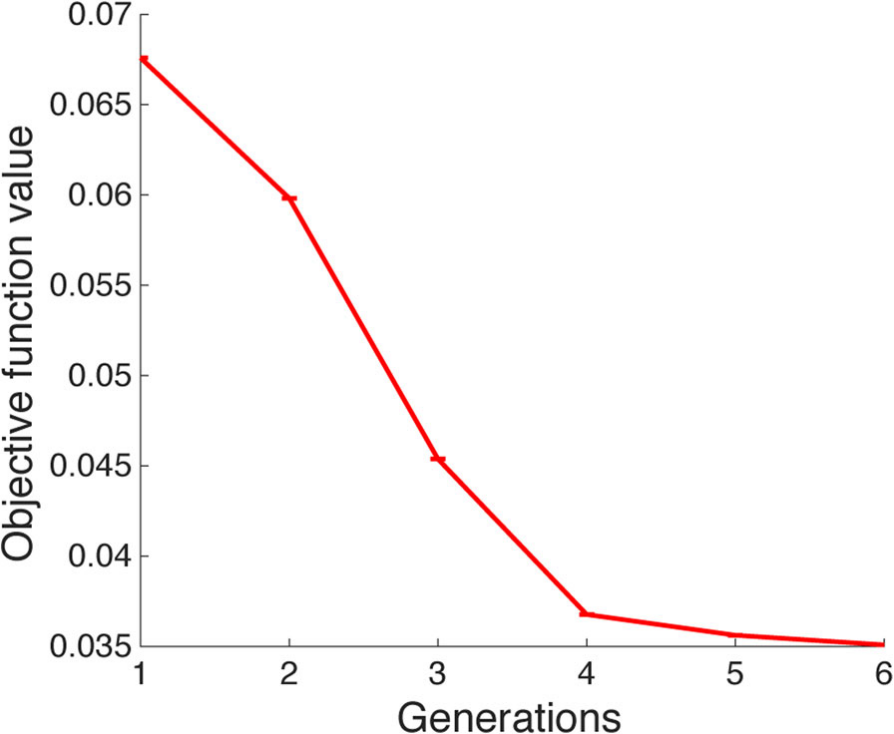
Genetic Algorithm performance. The deterministic precondition was introduced in a genetic algorithm strategy. The genetic algorithm was implemented using an adaptive population size using a population of 3000 individuals for the first generation, and a population of 20 individuals for the subsequent generations. As algorithm parameters we used *μ* = 0.2, and *ε* = 0.4. In the plot a decrease in the objective function value during the generations in the GA is shown

**Fig. 6:**
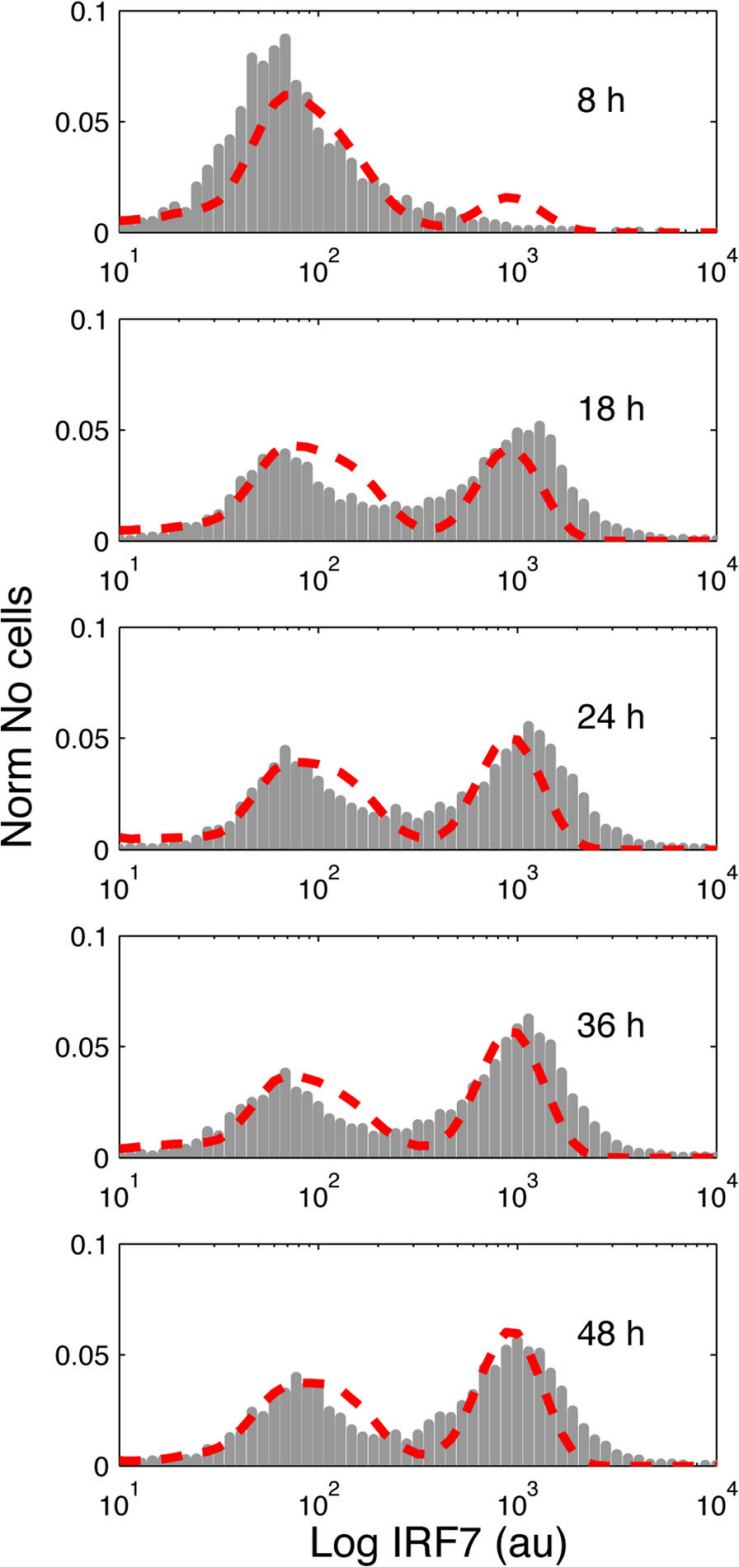
Comparison between the experimental data and stochastic simulations. Result of the fitting of the model with the experimental data using the genetic algorithm and the deterministic precondition. In the plots, the y-axis represents the normalized cell count and the x-axis represents the fluorescence quantity (arbitrary units, au) associated with the expression of the IRF7 protein. In gray we present the histograms that represent the experimental data, in red the PDF from the stochastic simulations

Then, we implemented the deterministic precondition using a genetic algorithm with adaptive population size. Here we implemented an initial population of 3000 individuals for the first generation, and 5 subsequent generations with 20 individuals. Notice, that a large initial population of parameters was needed by the expected high rejection percentage of parameters by the deterministic precondition during the first generation. As parameters for the algorithm we used *ε*=0.4 and *μ*=0.2. Our simulation results showed a constant decrease in the value of the objective function value during the generations, which indicates progress during fitting. By the use of the deterministic precondition 99% of the parameters were rejected in the first generation and in the subsequent generations around 30% of the parameter values were rejected, improving in this way the efficiency of the algorithm (see Fig. 5). The comparison between the experimental data and the model simulation distributions is given in Fig. 6 showing a high degree of agreement. In addition, to check the validity of the methodology further we fit the model to two additional flow cytometry measurements that describe the stimulation of the cell culture with 100U and 250U of IFN. The respective results are given in Additional file 6. Complete analysis of the performance of the use of the deterministic precondition in the genetic algorithm is given in Additional file 4.

### IRF7 temporal dynamics

**Fig. 7:**
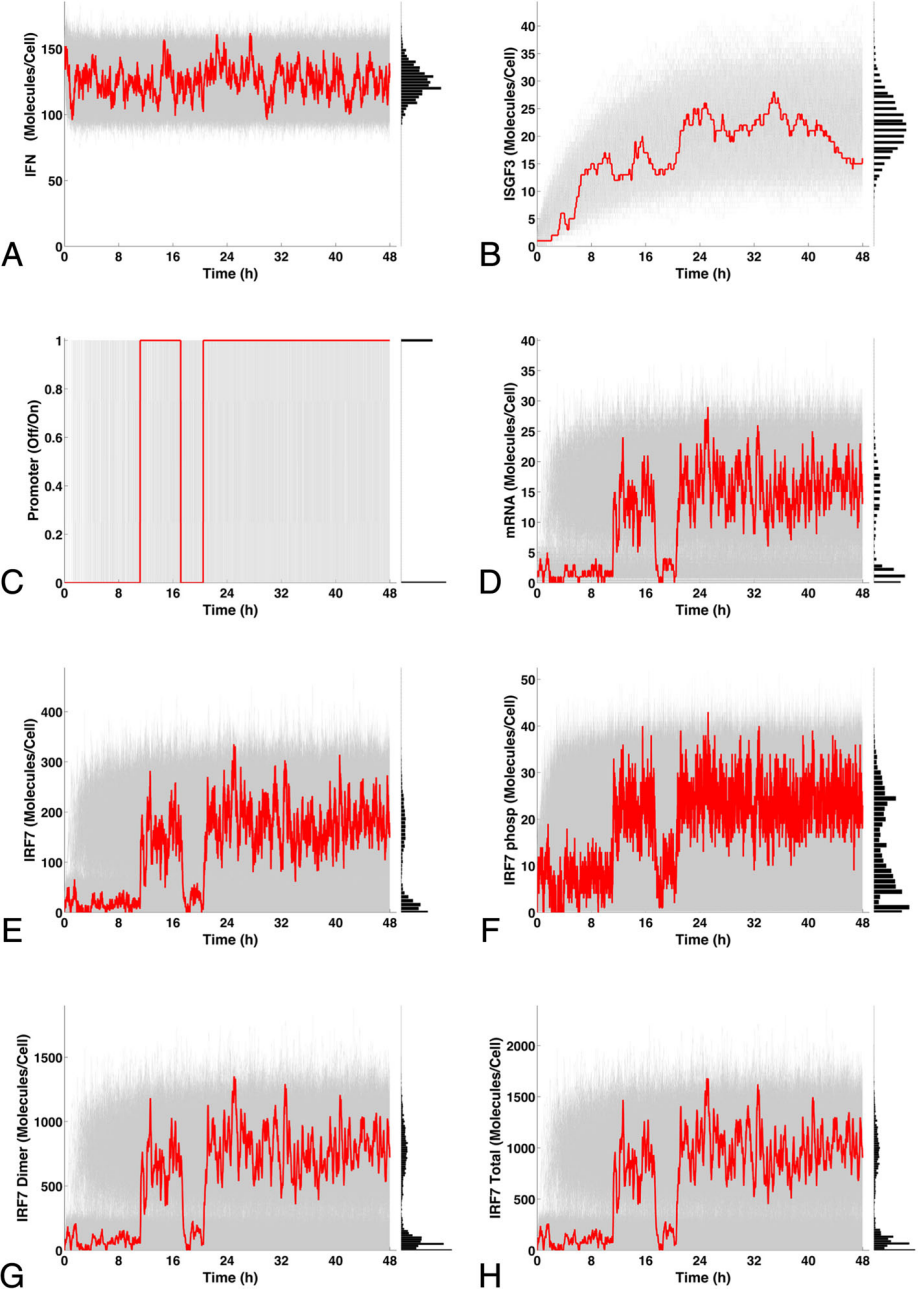
Time courses in the IRF7 circuit after IFN stimulation. The temporal dynamics of the promoter, mRNA and IRF7 protein dynamics were obtained after stochastic simulations. A representative trajectory that presents a single cell dynamics is given by the red lines. The stochastic simulations were repeated 1000 times using the same initial condition obtaining the histograms that represent the cell population. **a** Time courses of IFN showing the evolution to a steady state, the same is observed in **b** for the ISGF3. **c** IRF7 promoter shows the transition between the two possible states Off/On. **d** IRF7 mRNA showing a basal expression state and a state of active expression. Bimodality is observed in **e** for the IRF7 protein, the same bimodal behavior was observed in **f** for the IRF7 phosphorylated protein, in **g** for the IRF7 protein dimer, and in **h** for all forms of the IRF7 protein

The simple model of IRF7 gene expression described above is sufficient to explain IRF7 bimodality. Using the optimized parameter values given in Table 2 and the initial conditions given in Table 3, the stochastic temporal dynamics of the model were simulated and are given in Fig. 7. In Fig. 7-a it can be seen that the IFN concentration evolves to a steady state. Subsequently, the first affected variable is ISGF3 that equally evolves towards a stable steady state, see Fig. 7-b. Figure 7-c shows the IRF7 promoter dynamics exhibiting transitions between active/basal states. This promoter state transition is a characteristic of genes with regulated expression [43]. Subsequently, IRF7 mRNA expression displays a pattern that is affected by this stochastic switching between two possible states, one with basal expression and the other with active expression, see Fig. 7-d. For single-cell trajectories, IRF7 protein expression shows a switch-like expression. For the whole population of those trajectories bimodality is observed (Fig. 7-e), the same stands for the different forms of the IRF7 protein, phosphorylated (Fig. 7-f) dimer (Fig. 7-g), and the total amount of IRF7 proteins (Fig. 7-h). The system’s steady states are given in Table 4.

**Table 3:** System’s initial conditions

Variable	Initial condition (molecules/cell)
*IFN*	150
*I* *S* *G* *F*3	1
*Pa*	0
*mRNA*	1
*I* *R* *F*7	1
*I* *R* *F*7*p* *h* *o* *s* *p*	0
*I* *R* *F*7*d* *i* *m* *e* *r*	0

**Table 4:** System’s steady states

Variable	1^st^ sss (eigenvalue)	2^nd^ sss (eigenvalue)
*IFN*	0 (-15.95)	124.70 (-0.0017)
*I* *S* *G* *F*3	0 (-3.87)	22.00 (-0.049)
*Pa*	0 (-0.44)	0.99 (-0.072)
*mRNA*	1.20 (-0.071)	15.93 (-0.32)
*I* *R* *F*7	13.62 (-0.049)	180.32 (-0.44)
*I* *R* *F*7*p* *h* *o* *s* *p*	6.62 (-0.0017)	24.09 (-3.87)
*I* *R* *F*7*d* *i* *m* *e* *r*	60.19 (-0.0013)	796.69 (-58.03)
*I* *R* *F*7*t* *o* *t* *a* *l*	80.43	954.01

## Discussion

The promise of systems biology is to achieve a quantitative understanding of the molecular processes in the cell with the aid of computational models. However, a bottleneck is the availability of reliable parameter values needed in those models. Often, it is very difficult or even impossible to measure all of these parameters. For deterministic models, this problem has been well tackled by the development of efficient methods for parameter estimation. Contrarily, for stochastic dynamics the landscape of methods for parameter estimation is poorer and still under development. For this reason, innovative and efficient algorithms are needed to fit and validate realistic stochastic models.

During the last years important advances have been achieved in the field of parameter inference for stochastic models. On one hand, strategies that involve mathematical procedures of moment estimation have been suggested [17–19]. On the other, Bayesian methods that test multiple parameters values to find approximated solutions that represent the data have been successfully implemented [20]. Currently, the main problems observed in most methods include the computational cost, the accuracy of the obtained solution, and its potential to be implemented in large and non-linear systems. Different strategies have been suggested to improve the accuracy and alleviate the computational performance [21, 22].

In our method, we tackled the computational cost by introducing a deterministic precondition that works as a by-pass in the algorithm avoiding large amounts of unnecessary stochastic simulations. The concept of reducing the parameter space by introducing a precondition defined by the experimental data has been suggested previously by Hori et al. [21]. In their method, a small order linear model is used to optimize the experimental data by finding the root of a Lyapunov equation. In contrast, we use a deterministic precondition. Both approaches have advantages and disadvantages. Thus, Hori’s method is constrained by the need to find the Lyapunov equation. Our method is based on steady state calculation and Monte Carlo simulations that are standard and well-known methodologies used in systems biology.

Recently a new algorithm developed by Lillacci et al., has been shown to significantly reduce the computational cost and achieve a high accuracy fit for stochastic models and flow cytometry data [22]. This method uses the Kolmogorov distance as a metric to calculate the difference between model simulation and experimental data. By choosing this metric, it is possible to estimate the minimal number of simulations needed to compare experimental and model simulations under a certain tolerance value. This estimated value decreases as the number of experimental data increases. In typical flow cytometry experiments the number of measured cells is in the order of tens of thousands and this number in Lillacci’s Algorithm is translated in a reduction of at least one order of magnitude in the number of required stochastic simulations. This algorithm has been applied to a model with 18 reactions and 20 free parameters obtaining a good fit to flow cytometry experimental data that reproduces bimodal dynamics. Comparing our method with Lillacci’s algorithm it is important to point out that both methods tackle the computational cost issue in two fundamentally different ways. Lillacci’s algorithm minimizes the number of needed stochastic simulations, whereas our algorithm minimizes the number of parameters sets evaluated with stochastic dynamics. A powerful new algorithm may be the result of combining both methodologies.

It is well known from non-linear dynamics that the model architecture and parameter values determine the behavior of the system. Hence, more complex stable dynamics such as limit cycles, and higher-multistability (a system with more than two stable steady states) can be obtained. Unstable systems are usually not of interest in the biological context. Our method was designed and tested for monostable and bistable systems, all other cases being rejected by the deterministic precondition. The case of limit cycles can be better approached by existing parameter estimation methods for single time-course data [12–16]. The case of systems with higher-multistability can in principle be treated by our method, but the application is limited by the costly need to find multiple stable steady-states in the system. Finally, it is relevant to mention that comparing stable states of the ODE model with modes of the measured flow cytometry distribution in some models can present some inherent problems. Bimodal fluorescence distributions may have no relation to bistability, e.g. when the systems has large differences in transitions rates for the promoter states [44].

Our method was implemented using two well-established optimization strategies, random search and genetic algorithms. Using a random search algorithm we observed that less than 1% of the population of parameters are subject to stochastic evaluations, this reduction in the tested parameters allowed us to explore a larger proportion of the parameter space, which is especially relevant for systems with multiple unknown parameters and no initial parameter guesses. On the other hand, using genetic algorithms we achieved a convergence to a minimal value in the optimization function after a few generations (see Fig. 5). Additionally, during each generation in the genetic algorithm a large percentage of the total evaluated parameters is rejected by the deterministic precondition. This percentage is dependent on the parameter of the algorithm (rate of elitism and mutation rate). In both strategies the introduction of the deterministic precondition significantly improved the parameter estimation process. Moreover, the implementation of deterministic precondition is not restricted to random search and genetic algorithms, it can also be implemented in other optimization algorithms, e.g. particle swarm or simulated annealing. A complete analysis of the performance of using the deterministic condition is given in Additional file 4.

The accuracy of the method is given by the agreement between experimental data and simulations. As can be observed in Fig. 6 a good fit was found, albeit not a perfect one. We observed that even increasing the number of tested parameter values during the random search and/or increasing the number of generations during the genetic algorithm did not improve the fit. For this reason, we consider that the differences between the model and the experimental data might be explained by the fact that our system is a highly simplified model that was built by lumping some steps in the biological system. Our team is working to fit more complex models in a future publication. To test the reproducibility of the obtained parameter values, the method was repeated 100 times and distributions of the obtained parameters were built. As can be observed in Additional file 5, when a parameter is identifiable this is reflected in a narrow distribution, on the other hand, when a parameter is non-identifiable this is reflected in a wide distribution. In the example given by reactions (11) to (23) we observed that most parameters are not identifiable. Thus, non-identifiability is expected in the parameters contained in reactions (11) and (20). Those reactions have opposite effects on the IFN dynamics. For this reason, multiple combinations of values in parameters *V*_*IFN*_, *k*_*IFN*_ and *k*_*dIFN*_ have similar effects on the overall system dynamics. Contrarily, the parameters involved in the production of the mRNA (*k*_*Active*_ and *k*_*Basal*_) are better confined.

Rand et al. described bimodal gene expression of IRF7 after IFN stimulation. This phenomenon was explained at the cell population level by the effects of the IFN paracrine response [30]. Here we observed that bimodality also can be explained in absence of paracrine response and only taking into account the molecular mechanism responsible for the IRF7 promoter dynamics. Bistability in gene-expression circuits is commonly associated with the switching between active/basal states in the gene promoter. In most cases, DNA cooperativity in the promoter is the basis of promoter-state switching [45]. However, for type-I IFN responses a promoter activation in a cooperative manner has recently been discarded [46]. Taking the recent literature into account, we developed a model on the basis of a positive feedback loop circuit comprising the independent activation of ISRE and IRFE elements by one ISGF3 molecule and one IRF7 dimer, respectively (see Fig. 4). This model has the needed and sufficient elements to sustain a bistable system (that is a positive feedback loop and non-linear dynamics in the reaction rates) [47, 48]. Additionally, multiple model structures testing different biological scenarios including: additional feedback loops, additional intermediate elements, and promoter cooperative activation were tested, obtaining that the presented model reproduces the experimental data best. Our simulation results agree with the experimental data obtained from flow cytometry [30]. Additional characteristics observed in the experimental data are also reproduced by the model, such as the basal expression of IRF7 without IFN stimulation [29], see Additional file 7. The number of molecules in the active state of the system (given by the obtained 2 ^*nd*^ steady state, see Table 4) agrees with the order of magnitude reported for mammal mRNAs (average = 17 Molecules/Cell with a range between 1 to 200 Molecules/Cell) and the range of protein concentration (average = 50,000 Molecules/Cell with a range between 100 to 10^8^ Molecules/Cell) [49].

## Conclusion

Here, we present a method to fit stochastic models to experimental data. The method is based on the comparison of distributions. The central idea of the method is to use a deterministic precondition that is defined by the experimental data as a filter avoiding large amounts of costly stochastic simulations. Using this idea, the number of parameters evaluated under stochastic dynamics is reduced, resulting in a significant improvement in the performance of the algorithm. As a case study, we used a model of IRF7 gene expression investigating the origin of bimodality in its dynamics upon IFN stimulation. Our results allowed us to conclude that a circuit with IRF7 promoter activation by one IRF7 dimer and one ISGF3 molecule is sufficient to explain the observed bimodal dynamics.

## Additional files

Additional file 1 Pseudocode for the genetic algorithm with the deterministic precondition. Additional file with the pseudocode for the genetic algorithm. (PDF 85.9 kb)

Additional file 2 Identifying parameters for a constitutive gene expression circuit with in-silico data. Additional file with an example where the method is applied to a simulation case with synthetic data. The file contains **Figures A1-A3**, and **Tables A1** and **A2**. (PDF 930 kb)

Additional file 3 Random search strategy. Additional file with **Figure A4**. Results obtained by using the random search strategy. (PDF 930 kb)

Additional file 4 Algorithm’s speed and performance analysis. Additional file with an analysis of the algorithm’s speed and performance. The file contains **Figure A5**, and **Tables A3** and **A4**. (PDF 75.9 kb)

Additional file 5 Testing the reproducibility of the selected parameter values. Additional file containing **Figure A6** that shows the parameter distributions. (PDF 215 kb)

Additional file 6 Fitting the model to different doses of IFN. Additional file with figure A7 and A8 that shows the fits of the model to flow cytometry data describing the stimulation of cells with 100U and 250U of IFN. (PDF 188 kb)

Additional file 7 Model dynamics without IFN stimulation. Additional file with **Figure A9** that shows the time courses for the model in absence of IFN stimulation. (PDF 693 kb)

## Abbreviations

au: Arbitrary units
BAC: Bacterial artificial chromosome
DP: Deterministic precondition
GA: Genetic algorithm
IFN: Interferon
ISG: Interferon stimulated genes
ODE: Ordinary differential equation
PDF: Probability density function
RS: Random search
sss: Stable steady state
