# Supplementary material for: A new efficient approach to fit stochastic models on the basis of high-throughput experimental data using a model of IRF7 gene expression as case study
Source: BMC Syst Biol. 2017 Feb 20;11:26. doi: 10.1186/s12918-017-0406-4 (PMC5322793; doi:10.1186/s12918-017-0406-4)
Supplement: Additional file 1 — Pseudocode for the genetic algorithm with the deterministic precondition. Additional file with the pseudocode for the genetic algorithm. (PDF 85.9 kb) [file 12918_2017_406_MOESM1_ESM.pdf]

Additional File 1 — Pseudo-code for the genetic algorithm with the deterministic precondition

Data: High-throughput PDFs. Biochemical Model.
Define: Number of Free Parameters (θ_{fp}). Ranges for Parameter Values. Number of Generations (G). Population Size (PS). Mutation Rate (μ). Rate of Elitism (ϵ).
Result: Parameter values that best reproduce the experimental data.

GENERATE a initial population of random parameters.

```
for i = 1 : G do
    j = 1 ;
    while j < PS do
        Assign the j^th parameter set in the model;
        Run deterministic dynamics ;
        Test deterministic precondition ;
        if deterministic precondition is true then
            RUN stochastic simulations ;
            Objective Function (OF) evaluation ;
            fitness = 1/OF
        else
            Reject the j^th parameter set ;
            Set OF = ∞ ;
            fitness = 0;
        end
        j = j + 1
    end

    RANK individuals according to its fitness ;
    SELECT a number of parental individuals (PI), np = PS × ε ;
    RECOMBINE PI until generate a offspring number, no = PS − PI ;
    MUTATE each offspring with a number of mutations, nm = θ_fp × μ;

end
```

Algorithm A1: Genetic algorithm with a deterministic precondition
