# Supplementary material for: A new efficient approach to fit stochastic models on the basis of high-throughput experimental data using a model of IRF7 gene expression as case study
Source: BMC Syst Biol. 2017 Feb 20;11:26. doi: 10.1186/s12918-017-0406-4 (PMC5322793; doi:10.1186/s12918-017-0406-4)
Supplement: Additional file 2 — Identifying parameters for a constitutive gene expression circuit with in-silico data. Additional file with an example where the method is applied to a simulation case with synthetic data. The file contains Figures A1-A3, and Tables A1 and A2. (PDF 930 kb) [file 12918_2017_406_MOESM2_ESM.pdf]

Additional File 2 – Identifying parameters for a constitutive gene expression circuit with *in-silico* data.

The model describes the stochastic dynamics of two variables, protein and mRNA, of a gene with constitutive expression [1]. A graphical representation of the constitutive gene expression circuit is given in Fig 3-A in the main text. The model is described by the following reactions:

$$\varnothing \xrightarrow{\theta_1} mRNA, \tag{A1}$$

$$mRNA \xrightarrow{\theta_2} \varnothing, \tag{A2}$$

$$mRNA \xrightarrow{\theta_3} Protein, \tag{A3}$$

$$Protein \xrightarrow{\theta_4} \varnothing. \tag{A4}$$

Deterministic model

The corresponding deterministic model comprises the following ordinary differential equations (ODEs) (Eq. (A5) to (A6)) that describe the average dynamics of mRNA and protein.

$$\frac{mRNA}{dt} = \theta_1 - \theta_2 * mRNA, \tag{A5}$$

$$\frac{Protein}{dt} = \theta_3 * mRNA - \theta_4 * Protein. \tag{A6}$$

Steady state calculation.

$$Protein_{ss} = \frac{\theta_1 * \theta_3}{\theta_2 * \theta_4} \tag{A7}$$

$$mRNA_{ss} = \frac{\theta_1}{\theta_2} \tag{A8}$$

Evaluating the Jacobian at the steady states, we get:

$$J = \begin{bmatrix} \frac{\partial(\theta_1 - \theta_2 * mRNA)}{\partial mRNA} & \frac{\partial(\theta_1 - \theta_2 * mRNA)}{\partial Protein} \\ \frac{\partial(\theta_3 * mRNA - \theta_4 * Protein)}{\partial mRNA} & \frac{\partial(\theta_3 * mRNA - \theta_4 * Protein)}{\partial Protein} \end{bmatrix} \tag{A9}$$

Evaluating the Jacobian at the equilibrium point:

$$J = \begin{bmatrix} -\theta_2 & 0 \\ \theta_3 & -\theta_4 \end{bmatrix} \tag{A10}$$

And the Eigenvalues are the solution of the equation:

$$|\lambda \mathbf{I} - \mathbf{J}| = 0 \tag{A11}$$

that is:

$$\begin{bmatrix} \lambda + \theta_2 & 0 \\ \theta_3 & \lambda + \theta_4 \end{bmatrix} = (\lambda + \theta_2)(\lambda + \theta_4) = 0 \tag{A12}$$

By solving the previous equation for λ we get:

$$\lambda_1 = -\theta_2 \tag{A13}$$

$$\lambda_2 = -\theta_4 \tag{A14}$$

According to stability theory [2], given that the obtained Eigenvalues are negative the equilibrium point (or steady state) is stable.

Calculating the minimal number of stochastic simulations.

Experimental data distributions came from measurements of tens of thousands of single cells by flow cytometry, whereas distributions from the stochastic model requires ns stochastic simulations. Computing the model distribution using ns in the order of tens of thousands is computationally expensive even for simple models. To reduce computational cost in our simulations we calculated a minimal number of stochastic simulations $\hat{ns}$ needed to build the distribution with a quality good enough to be used during the optimization strategy.

To calculate $\hat{ns}$ the following empirical procedure was applied: First, we built probability density functions (PDFs) from *in-silico* data (P_{is}) using the model described by reactions (A1) to (A4) with a set of known parameter values $\theta^{(o)}$ and by running 10000 stochastic simulations. Then, using the same model and $\theta^{(o)}$ we tested different values for ns to built candidate PDFs (P_s). To measure the similitude between P_s and P_{is} we used the objective function given by Eq.(2) from the main text. As can be observed in Fig A1-A) using a large value for ns reduces the value of the objective function. Nevertheless, using a larger value for ns is reflected in a larger computational cost, see Fig A1-B). Therefore, a balance between accuracy and computational cost was determined where no qualitative differences was observable between P_s and P_{is}. By using values for ns larger than 1000 no differences were observable between P_s and P_{is}, see Fig A2). With this we defined a value for $\hat{ns} = 1000$. This number is in concordance to previous works where the minimal number of stochastic simulations used to compare experimental and simulation distribution was calculated by using the properties of the Kolmogorov distribution in Lillacci's algorithm [3]. It is important to mention that in Lillacci's algorithm this number has been proved to only depend on the number of cell measurements from the experimental data.

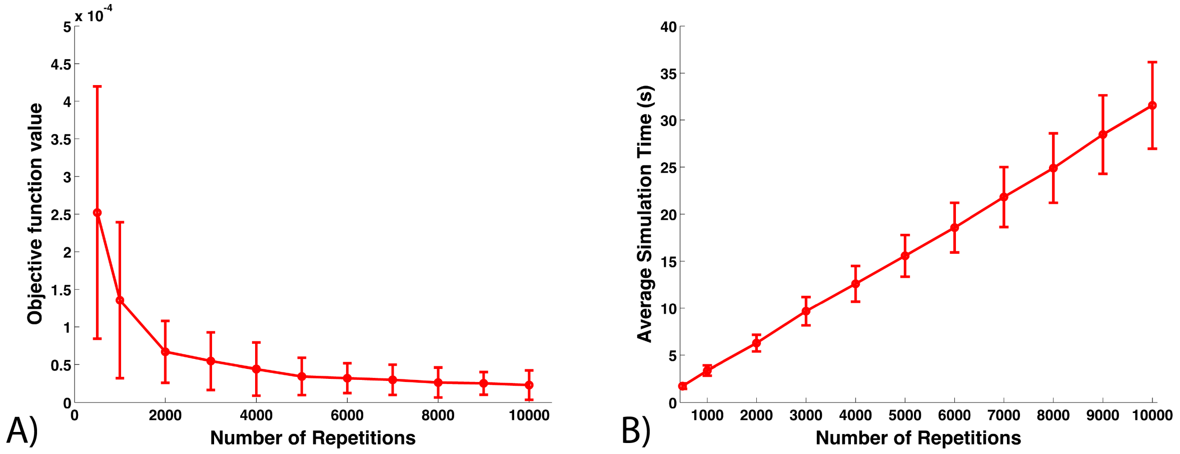

Figure A1: **Minimal Number of simulations vs Simulation Time.** A) Objective function value obtained by comparing the *in-silico* data distributions vs those obtained by varying the ns values. B) Average simulation time needed to solve the stochastic system using different values for ns. Three independent repetitions of the experiments were performed and those results are considered in the error bars.

In addition to the previous empirical procedure, we used the two-sample Kolmogorov-Smirnov hypothesis test to evaluate if the data used to build P_s with different ns are different from the data used to build P_{is}. This test was implemented in Matlab. With this, we tested the null hypothesis $P_s = P_{is}$, using different values of ns. The result of the test (H) is 1 if the test rejects the null hypothesis at the 5% significance level, and 0 otherwise. The result is given in Table A1 and it can be seen that for $ns \geq 500$ the null hypotheses are accepted. In contrast, we obtained that for less than 500 repetitions the null hypothesis was rejected at some time points (data not shown). Even though, $ns = 500$ gives the minimal number of repetitions where H is rejected, its observed variability is very high (see Fig A1-A). For this reason, we selected $\hat{ns} = 1000$ as a valid number to perform computations.

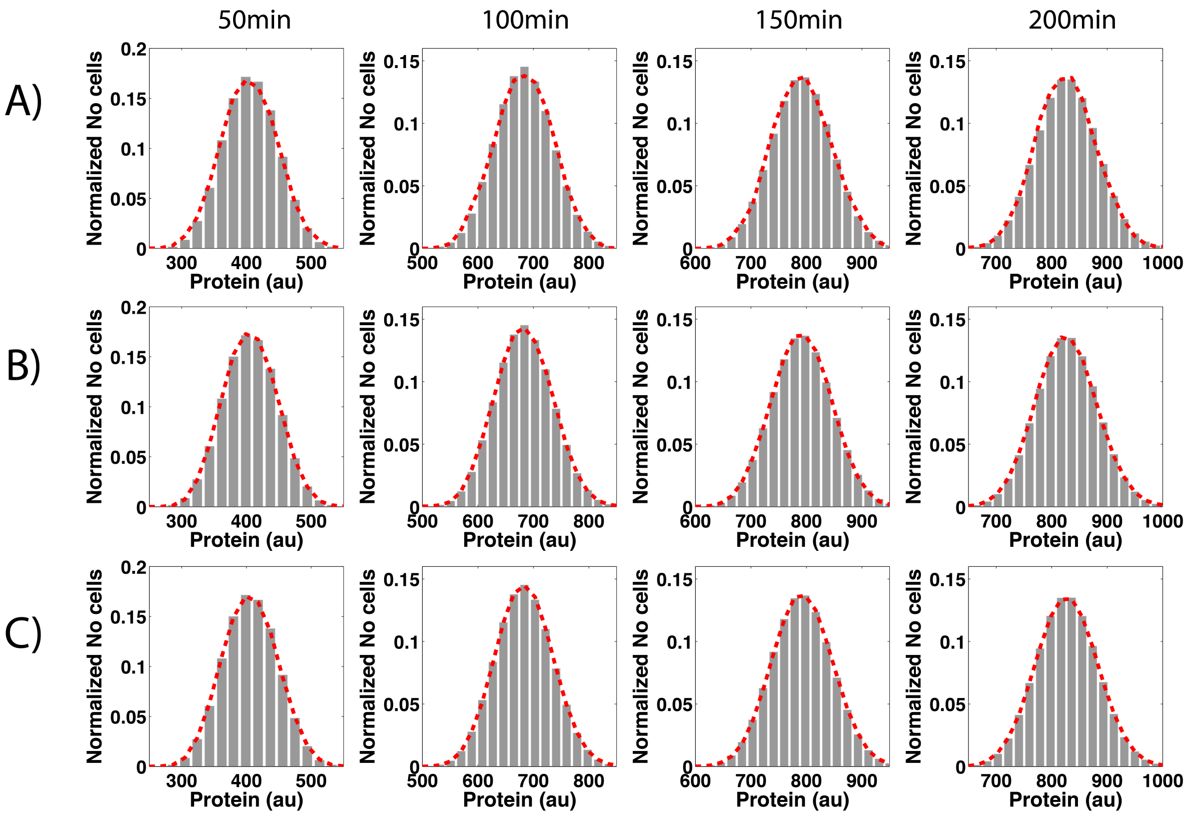

Figure A2: **Comparing distributions made with different repetitions.** Using the system given by reactions (A1) to (A4) and $\theta^{(o)}$ PDFs were build using different numbers of stochastic simulations. A) 500 repetitions. B) 1000 repetitions. C) 10000 repetitions. In gray the histograms from the synthetic data are given, in red the simulated PDF.

Table A1: **p-value obtained from the Two-sample Kolmogorov-Smirnov test between synthetic data and reference data varying ns.**

ns	t_1		t_2		t_3		t_4	
	H	p-value	H	p-value	H	p-value	H	p-value
**500**	0	0.8192	0	0.6558	0	0.9837	0	0.6743
**1000**	0	0.9761	0	0.8227	0	0.5702	0	0.3981
**10000**	0	0.9520	0	0.8111	0	0.7209	0	0.5206

$H = 1$, if the test rejects the null hypothesis at the 5% significance level, and 0 otherwise. $t_1 = 50$ min, $t_2 = 100$ min, $t_3 = 150$ min and $t_4 = 200$ min.

Accuracy of the method.

To test the accuracy of the new method we generated *in-silico* data by simulating the model described by reactions (A1) to (A4) and using a set of known parameter values $\theta^{(o)}$. Multiple parameter estimates $\hat{\theta}$ were used to run stochastic simulations from which PDFs were computed. After implementing our method n_e times, the accuracy of the result was measured by calculating the average (av), standard deviation (sd), and the average relative error (ARE).

Table A2: **Method accuracy.**

**Metric**	$\theta_1^o = 5$	$\theta_3^o = 0.1$
$av \pm sd$	4.72 ± 0.53	0.11 ± 0.013
ARE	0.0925	0.10

Where the *av* was calculated as:

$$av = \frac{1}{n_e} \sum_{i=1}^{n_e} \hat{\theta}^{(i)}, \tag{A15}$$

and the *ARE* was calculated as:

$$ARE = \frac{1}{n_e} \sum_{i=1}^{n_e} \frac{|\hat{\theta}^{(i)} - \theta^{(o)}|}{\theta^{(o)}}. \tag{A16}$$

The central idea behind the use of the deterministic precondition is to reduce the computational time by only testing parameter values with good chances to reproduce the experimental data. In the *in-silico* case the true parameters were known. The parameter space presented in Fig A3 was sampled by generating 10000 random parameter sets (black dots). The true parameter values are represented by the intersection of the two red lines. By applying the deterministic precondition only a subsets of parameters were accepted (magenta dots), this represents the 3.1% of the original parameters that were evaluated under stochastic dynamics, reducing in this way the total simulation time. Notice that the area with the true parameters is contained by the parameters that fulfill the deterministic precondition.

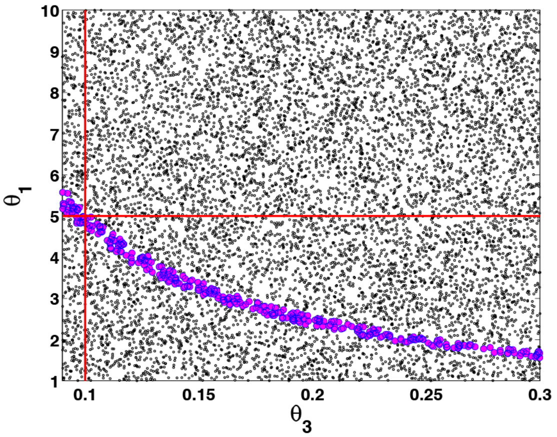

Figure A3: **Parameter space of a constitutive gene expression model.** The parameter space of the system given by reactions (A1) to (A4). The parameter space was sampled using a random combination of parameters inside a selected range (black dots). True parameters are represented by the intersection of the red lines, and parameters that fulfill the deterministic precondition are represented by magenta dots.

References

Stefan Legewie, Hanspeter Herzel, Hans V Westerhoff, and Nils Blüthgen. Recurrent design patterns in the feedback regulation of the mammalian signalling network. *Molecular systems biology*, 4(1):190, 2008.

Steven H Strogatz. Nonlinear dynamics and chaos with applications to physics, biology, chemistry, and engineering. *Westview Press*, 2000.

Gabriele Lillacci and Mustafa Khammash. The signal within the noise: efficient inference of stochastic gene regulation models using fluorescence histograms and stochastic simulations. *Bioinformatics*, 29(18):2311–2319, 2013.
