# Supplementary material for: A new efficient approach to fit stochastic models on the basis of high-throughput experimental data using a model of IRF7 gene expression as case study
Source: BMC Syst Biol. 2017 Feb 20;11:26. doi: 10.1186/s12918-017-0406-4 (PMC5322793; doi:10.1186/s12918-017-0406-4)
Supplement: Additional file 3 — Random search strategy. Additional file with Figure A4. Results obtained by using the random search strategy. (PDF 930 kb) [file 12918_2017_406_MOESM3_ESM.pdf]

Additional File 3 – Random search strategy.

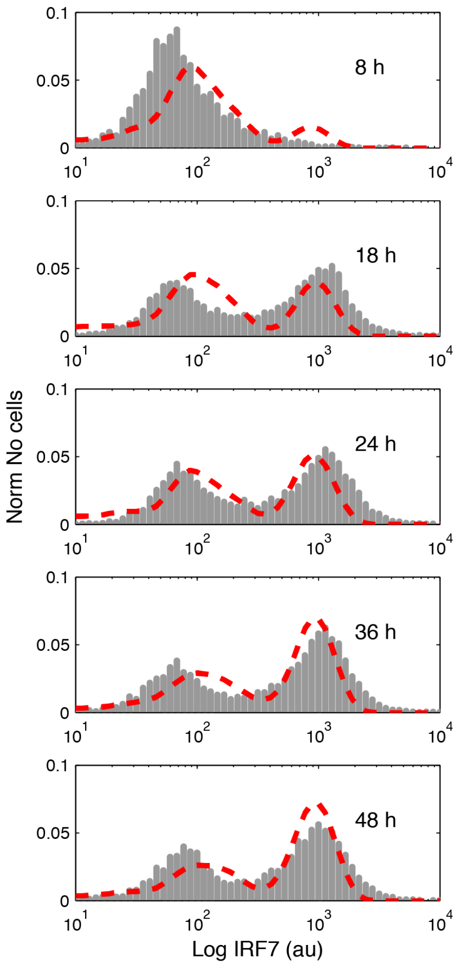

Figure A4: **Fitting of the model with the experimental data by the random search strategy.** Result of the fitting of the model to the experimental data. 10000 random parameter sets were evaluated under deterministic dynamics. Less than the 1% of those parameter values passed the deterministic precondition and were evaluated under stochastic dynamics. The parameter set that gives the minimal value in the objective function evaluation was plotted. In the plots, the y-axis represents the normalized cell count and the x-axis represents the fluorescence quantity (arbitrary units, au) associated with the expression of the IRF7 protein. In gray we present the histograms that represent the experimental data, in red the PDF obtained using the suggested optimization algorithm.
