# Supplementary material for: A new efficient approach to fit stochastic models on the basis of high-throughput experimental data using a model of IRF7 gene expression as case study
Source: BMC Syst Biol. 2017 Feb 20;11:26. doi: 10.1186/s12918-017-0406-4 (PMC5322793; doi:10.1186/s12918-017-0406-4)
Supplement: Additional file 4 — Algorithm’s speed and performance analysis. Additional file with an analysis of the algorithm’s speed and performance. The file contains Figure A5, and Tables A3 and A4. (PDF 75.9 kb) [file 12918_2017_406_MOESM4_ESM.pdf]

Additional File 4 – Algorithm's speed and performance analysis.

Execution time

All simulations were performed using a computer that had Core i7 2GHz with 8 GB of memory. The software was coded in Matlab and parallel computing was used allowing up to 8 independent evaluations at the same time. The deterministic precondition (DP) was implemented using two standard optimization strategies, random search (RS) and genetic algorithm (GA).

In the first implementation the DP was introduced in a RS optimization strategy. For RS implementation different sets of random parameter sets were generated using the ranges given in Table 2 from the main text. Three independent optimization runs and the mean and standard deviation (sd) are given for the simulation time, the percentage of parameters that were evaluated under stochastic dynamics and the final value for the objective function (OF).

Table A3: **Random Search Performance.**

Strategy	Parameter sets	Time (min)	% Evaluations	OF
**RS + DP**	10000	151.13 ± 8.85	0.24±0.0 %	0.0378 ± 0.0016

Results are given as mean ± s.d. of three independent runs.

The deterministic precondition was introduced in a GA strategy. In our system we used an GA with adaptive population size, here we implemented a population of 3000 individuals for the first generation, and 5 subsequent generations with 20 individuals. The execution time in the GA was calculated using a mutation rate ($\mu = 0.2$) and a rate of elitism ($\epsilon = 0.4$).

Table A4: **Genetic Algorithm Performance.**

Strategy	Time (min)	% Eval 1st Gen	% Evaluations	OF
**GA + DP**	282.41 ± 59.68	0.3±0.0%	59±4%	0.0371 ± 0.0005

Results are given as mean ± s.d. of three independent runs.

Computational efficiency of the algorithm using the deterministic precondition.

To determine the computational time to perform the parameter estimation using the DP, we compared the computational time taken to estimate parameters using the RS and GA with the DP vs. the estimated simulation time taken by RS and GA without the DP. The estimated simulation time was calculated as the average simulation time taken by the evaluation of one parameter set, that is 19.49 min (from a total of 100 random parameter sets), then this number was multiplied by the total parameters needed to complete the RS and the GA, 10000 and 3100 parameters, respectively.

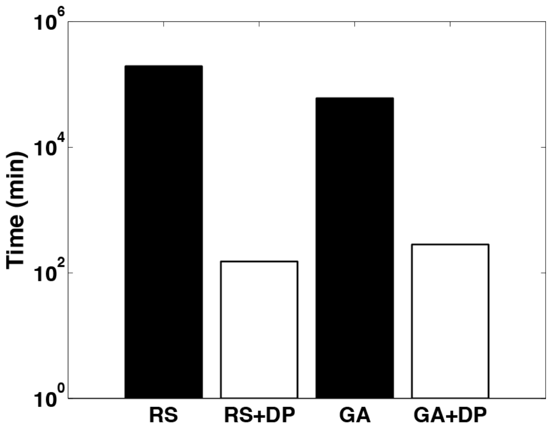

Figure A5: **Computational efficiency of the algorithm using the deterministic precondition.** Comparison between the computational time between the RS and GA with and without the DP. In white is given the computational time for the RS and GA with the DP. In black is given the estimated computational time for the RS and GA without the DP. To calculate a theoretical simulation time for the RS and GA without using the deterministic precondition we estimated the average simulation time for one parameter set and then this number was multiplied by the total number of parameters evaluated during the RS or during each generation in the GA.
