# Supplementary material for: A new efficient approach to fit stochastic models on the basis of high-throughput experimental data using a model of IRF7 gene expression as case study
Source: BMC Syst Biol. 2017 Feb 20;11:26. doi: 10.1186/s12918-017-0406-4 (PMC5322793; doi:10.1186/s12918-017-0406-4)
Supplement: Additional file 5 — Testing the reproducibility of the selected parameter values. Additional file containing Figure A6 that shows the parameter distributions. (PDF 215 kb) [file 12918_2017_406_MOESM5_ESM.pdf]

Additional File 5 – Testing the reproducibility of the selected parameter values.

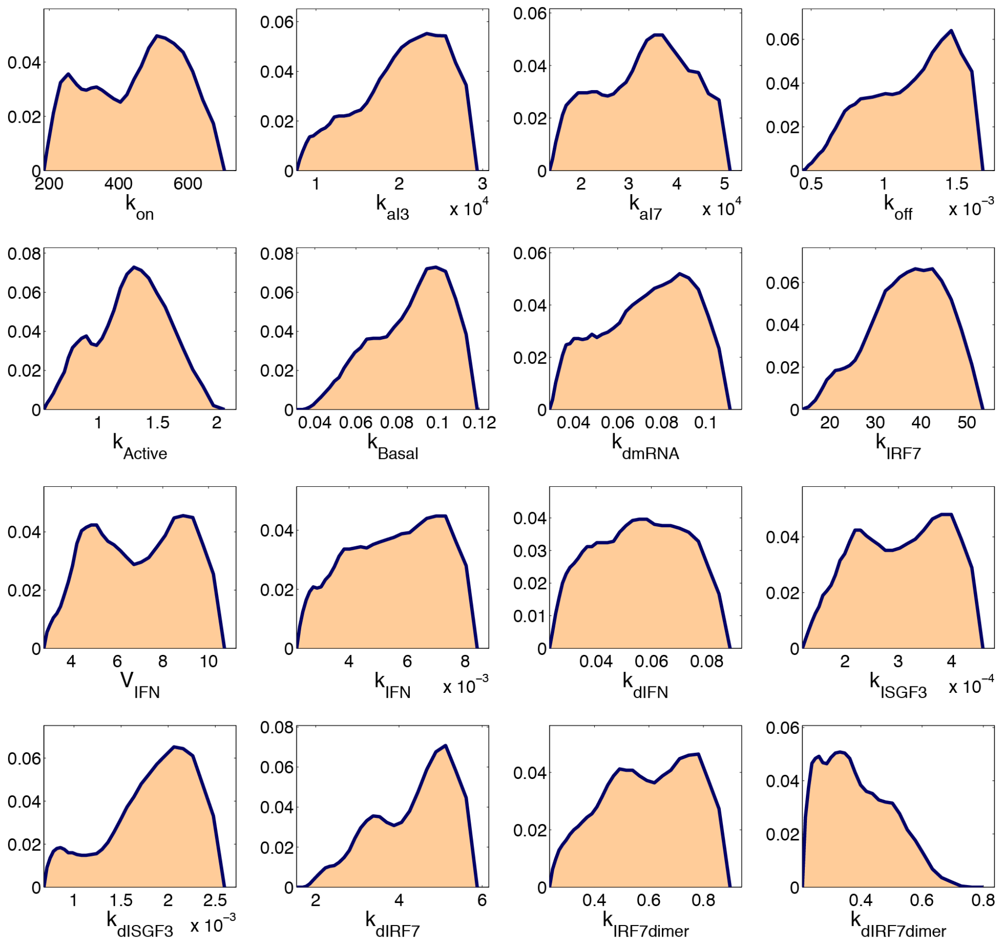

Figure A6: **Distributions for the 16 parameter values for the IRF7 model.** Using the IRF7 model given by reactions (11) to (23), and the parameter ranges given in Table 2 from the main text. The distributions were calculated by running 100 independent random searches with 10000 parameters each one. The parameter used for the distribution were selected by the algorithm, with values for $O.F. < 0.04$, which represents a good fit to the experimental data.
