# Supplementary material for: A new efficient approach to fit stochastic models on the basis of high-throughput experimental data using a model of IRF7 gene expression as case study
Source: BMC Syst Biol. 2017 Feb 20;11:26. doi: 10.1186/s12918-017-0406-4 (PMC5322793; doi:10.1186/s12918-017-0406-4)
Supplement: Additional file 6 — Fitting the model to different doses of IFN. Additional file with figure A7 and A8 that shows the fits of the model to flow cytometry data describing the stimulation of cells with 100U and 250U of IFN. (PDF 188 kb) [file 12918_2017_406_MOESM6_ESM.pdf]

Additional File 6 – Fitting the model to different doses of IFN.

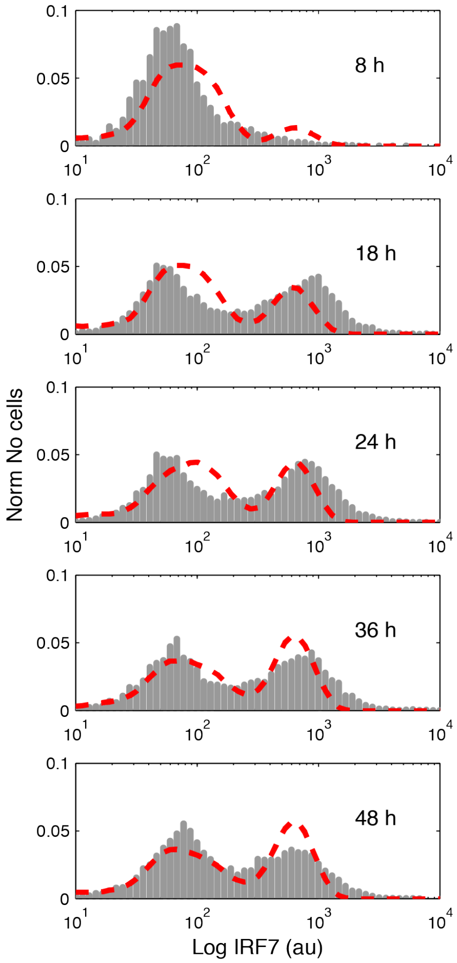

Figure A7: **Fitting of the model to flow cytometry data describing the stimulation of cells with 100U of IFN.** Result of the fitting of the model with the experimental data using the genetic algorithm and the deterministic precondition. In the plots, the y-axis represents the normalized cell count and the x-axis represents the fluorescence quantity (arbitrary units, au) associated with the expression of the IRF7 protein. In gray we present the histograms that represent the experimental data, in red the PDF from the stochastic simulations. Initial conditions were kept as in Table 3 from the main text, except the IFN variable was set to 100 Molecules/Cell.

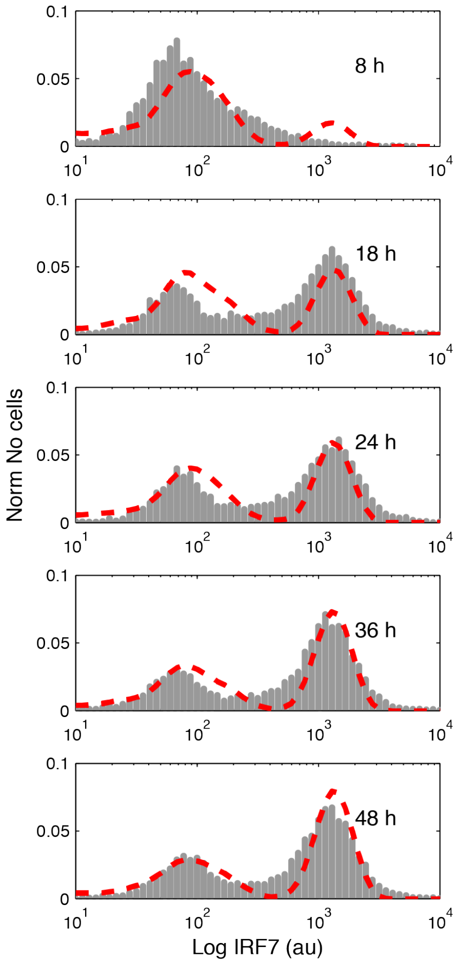

Figure A8: **Fitting of the model to flow cytometry data describing the stimulation of cells with 250U of IFN.** Result of the fitting of the model with the experimental data using the genetic algorithm and the deterministic precondition. In the plots, the y-axis represents the normalized cell count and the x-axis represents the fluorescence quantity (arbitrary units, au) associated with the expression of the IRF7 protein. In gray we present the histograms that represent the experimental data, in red the PDF from the stochastic simulations. Initial conditions were kept as in Table 3 from the main text, except the IFN variable was set to 250 Molecules/Cell.
