# Supplementary material for: A new efficient approach to fit stochastic models on the basis of high-throughput experimental data using a model of IRF7 gene expression as case study
Source: BMC Syst Biol. 2017 Feb 20;11:26. doi: 10.1186/s12918-017-0406-4 (PMC5322793; doi:10.1186/s12918-017-0406-4)
Supplement: Additional file 7 — Model dynamics without IFN stimulation. Additional file with Figure A9 that shows the time courses for the model in absence of IFN stimulation. (PDF 693 kb) [file 12918_2017_406_MOESM7_ESM.pdf]

Additional File 7 – Model dynamics without IFN stimulation.

To mimic a population of cells without IFN stimulation the model was simulated using an initial concentration of IFN equal to zero. From the simulations it can be observed that in absence of IFN stimulation the promoter shows no transitions to the Pa state. A small production of mRNA and IRF7 protein is observed, which is given by the small rate of mRNA synthesis in a promoter in basal state.

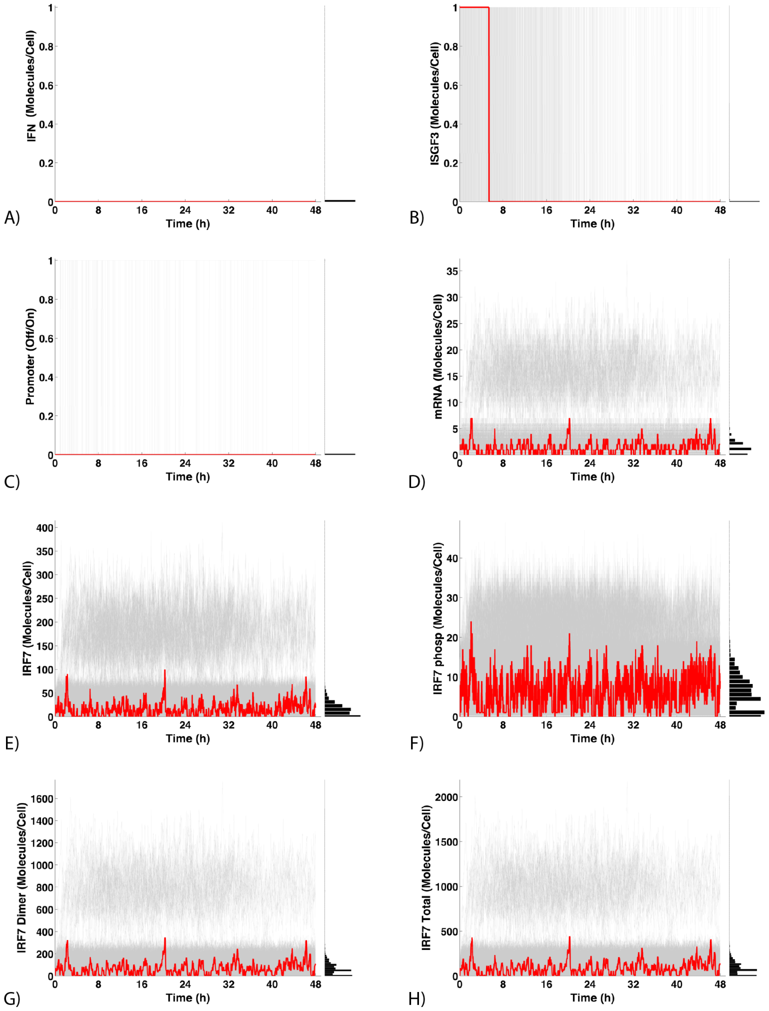

Figure A9: **Time courses in the IRF7 circuit without IFN stimulation.** The temporal dynamics of the promoter, mRNA and IRF7 protein dynamics were obtained after stochastic simulations. A representative trajectory that presents a single cell dynamics is given by the red lines, the stochastic simulations were repeated 1000 times using the same initial condition obtaining the histograms that represent the cell population. A constant state is observed in A) for the IFN, in B) for the ISGF3, and in C) for the IRF7 promoter. A basal expression is observed in D) for the IRF7 mRNA, in E) for the IRF7 protein, in F) for the IRF7 protein phosphorylated, in G) for the IRF7 protein dimer, and in H) for all forms of the IRF7 protein.
